# Creating Linkage Permutations to Prevent Self-Intersection and Enable Deployable Networks of Thick-Origami

**DOI:** 10.1038/s41598-018-31180-4

**Published:** 2018-08-28

**Authors:** Alden Yellowhorse, Robert J. Lang, Kyler Tolman, Larry L. Howell

**Affiliations:** 1 Dept. Mechanical Engineering, Brigham Young University, Provo, UT 84602 USA; 2 Lang Origami, Alamo, CA 94507 USA; 3 Toyota Motor North America, Saline, MI 48176 USA

## Abstract

Origami concepts show promise for creating complex deployable systems. However, translating origami to thick (non-paper) materials introduces challenges, including that thick panels do not flex to facilitate folding and the chances for self-intersection of components increase. This work introduces methods for creating permutations of linkage-based, origami-inspired mechanisms that retain desired kinematics but avoid self-intersection and enable their connection into deployable networks. Methods for reconfiguring overconstrained linkages and implementing them as modified origami-inspired mechanisms are proved and demonstrated for multiple linkage examples. Equations are derived describing the folding behavior of these implementations. An approach for designing networks of linkage-based origami vertices is demonstrated and applications for tessellations are described. The results offer the opportunity to exploit origami principles to create deployable systems not previously feasible.

## Introduction

Origami-inspired mechanisms have been demonstrated to hold significant potential for solving a variety of engineering problems^1^, including the deployment of antennas^2^ and solar panels^3^, shelters^4^, radiators for spacecraft^5^, surgical equipment^6^, nanomachines^7–9^, robotics^10,11^ electronics^12^ and airbag stowage^13^. However, one barrier preventing origami from being fully utilized involves successfully including thickness in the mechanism^14^. While thin origami models may deform to accommodate their small thickness during folding^15^, thicker materials make this difficult or impossible. Furthermore, thickness presents increased challenges for self-intersection of components. Consequently, developing methods for creating thick, origami-inspired mechanisms is essential for exploiting the performance benefits of origami-based structures.

An important part of designing thick origami mechanisms is preventing distortions during the folding process. This ability to fold without distorting the panels is called rigid-foldability^16^. While it is possible to design thick origami that is rigid-foldable^17^, the thickness of the material complicates the process because it often requires the modification of the crease pattern to retain mobility. Linkage-based origami has been demonstrated as one way to address this^18–20^ through a variety of different linkage-based models^21^. Deployable networks of linkages have also been investigated^22–26^. Both Tachi^27^ and Lang and Howell^28^ studied problems in the design of zero-thickness, rigid-foldable, quadrilateral meshes. This work has been supplemented by research on thick tessellations^29–33^ and 3D, thin tessellations^34,35^. Nevertheless, there is still much to learn about how to modify these mechanisms for use in materials other than paper. Processes for connecting origami-inspired linkages into networks that can be used in larger deployable mechanisms would also be helpful.

The objective of this paper is to enable translation of origami to thick materials by developing methods that generate permutations of spatial linkages with desirable characteristics. These characteristics include no self-intersection, maintaining the same number of degrees of freedom as the root linkage, and the existence of a simple kinematic model, which is particularly important for mechanisms based on spatial linkages. Hinge transposes, thickness shifts, and splitting vertices are proposed as ways to generate linkage permutations with these characteristics. These methods will help create modified spatial linkages that are suitable for use in linkage tessellations.

## Results

**Figure 1:**
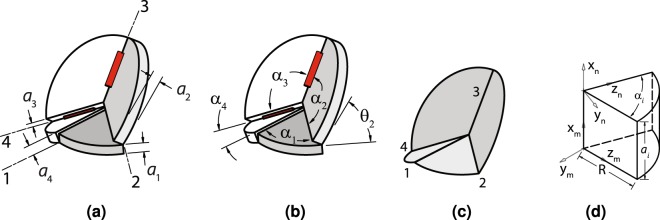
Linkage geometry in origami-inspired mechanisms. The Bennett linkage described with (**a**) thicknesses *a*_*i*_ and hinges indicated by numbers 1 through 4 and (**b**) angles *α*_*i*_ and *θ*_*i*_. A zero-thickness equivalent (**c**) where *a*_*i*_ = 0 and two frames *m* and *n* are used to define a panel (d) where *m* and *n* are related by a homogeneous transformation *T*.

The unique kinematics of overconstrained linkages allow them to be implemented as origami-inspired mechanisms because their hinge axes are not parallel. This is illustrated through the Bennett linkage with the joints and thicknesses labeled in Fig. 1a and the sector angles noted in Fig. 1b. Here, it can be seen that joint axes 1, 2, 3 and 4 are neither parallel nor co-planar. The similarity between this linkage and an origami vertex can be seen as *a*_*i*_ = *d*_*i*_ = 0 for a zero-thickness vertex in Fig. 1c. Multiple variations of this and other linkages can be obtained through three different methods presented in this section. Proving that these methods do not prevent the mechanism from moving is possible if we represent the linkage using the Denavit-Hartenburg (DH) convention.

The DH convention^36^ uses four parameters (*θ*, *a*, *d* and *α*) in a homogeneous transformation matrix *T*_*i*_(*θ*_*i*_, *a*_*i*_, *d*_*i*_, *α*_*i*_) to describe each link. *T*_*i*_ is defined in the Supplementary Information (SI) and represents a set of consecutive, specific rotations and translations that relate frames attached to two adjacent links. Because it must be true that the product of all *T*_*i*_ in a linkage loop must be the identity matrix, a formal definition of a linkage can be made for this work.

### **Definition 1**

*A mechanism consisting of a closed loop of n links is defined as a hooplinkage if and only if*

1 $${T}_{1}{T}_{2}\ldots {T}_{n}=\prod _{i=1}^{n}\,{T}_{i}=I$$

*where each T*_*i*_ *relates two frames rigidly fixed to adjacent links*. *Only θ*_*i*_ *in each T*_*i*_ *is allowed to vary*.

This definition has been used to derive formulas governing the behavior of multiple hoop linkages such as the Bennett linkage, as listed in the SI.

In thick origami, the fold angle, *θ*, the panel sector angle, *α*, and the panel thickness, *a*_*i*_, as shown in Fig. 1 are key variables describing the vertex geometry. They are naturally described by the twist parameter *α*_*i*_ and linear offset *a*_*i*_ in the DH representation. The parameter *d* is usually zero in these models. Using this transform and the frames shown in Fig. 1d, we define an origami panel that depends on the transform and has a simple shape.

### **Definition 2**

*Let two hinge axes z*_*m*_ *and z*_*n*_ *be related by a DH transformation T defined by the parameters θ*, *a*, *d*, *α*. *Also*, *let* ${\hat{z}}_{m}$, ${\hat{x}}_{m}$ *and* ${\hat{z}}_{n}$ *represent unit vectors aligned with the z- and x-axes of frame m and the z-axis of frame n*, *respectively*. *The panel P is defined by the set of all points satisfying*

2 $$P=\{h\in {{\mathbb{R}}}^{3}|h=p{\hat{z}}_{m}+q{\hat{z}}_{n}+v{\hat{x}}_{m}\}$$

*with respect to frame m where* 0 < *p* < *R*, 0 < *q* < *R*, 0 < *v* < *a*, *and R is a scalar that defines the curved boundary of the panel shown in* Fig. 1d.

### Hinge Transpose

**Figure 2:**
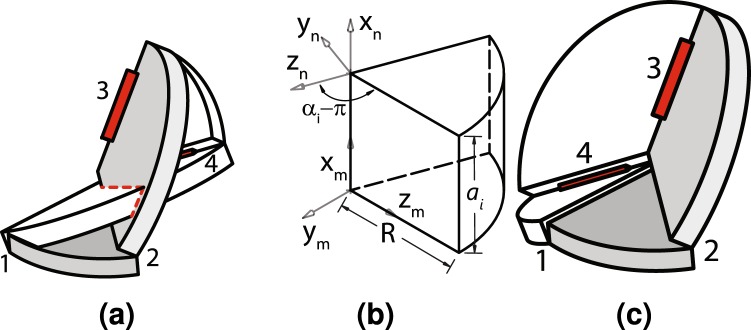
Hinge transpose. A Bennett linkage with self-intersection (**a**), a transposed panel (b) and the same linkage with a transpose of the fourth hinge (**c**) that produces a developable version of the mechanism. The unchanged links are in gray.

Here, we introduce the concept of a hinge transpose. It will be valuable in preventing self-intersection of links as shown in Fig. 2a, enabling new configurations of mechanisms. This transpose takes place when the twist angles *α* of the two links adjacent to the hinge are replaced by their negative supplements (e.g. *α* − *π*) as shown in Fig. 2b. This transpose is shown in Fig. 2c. While hinges 1, 2, and 3 remain in the same position, the fourth hinge changes direction. A hinge transpose can also be more formally defined below.

#### **Definition 3**

*Given a hoop linkage B composed of n homogeneous transformations T*_*i*_, *a hinge transpose of a joint between the i-th and (i* + *1)-th link consists of replacing transforms i and i* + *1 with transforms* ${T}_{i}^{^{\prime} }$ and ${T}_{i+1}^{^{\prime} }$ where ${T}_{i}\ne {T}_{i}^{^{\prime} }$ and ${T}_{i+1}\ne {T}_{i+1}^{^{\prime} }$.

This definition leads to the following theorem and corollary that such a transformation will not prevent the resulting mechanism from moving. The fact that multiple hinge transposes also do not prevent movement of the mechanism can be proved through variable substitutions, as shown in the SI.

#### **Theorem 1**

*If we define a specific hinge transpose which exchanges T*_*i*_ = *T*_*i*_(*θ*_*i*_, *a*_*i*_, *d*_*i*_, *α*_*i*_) *and T*_*i*+1_ = *T*_*i*+1_(*θ*_*i*+1_, *a*_*i*+1_, *d*_*i*+1_, *α*_*i*+1_) for ${T}_{i}^{^{\prime} }$ and ${T}_{i+1}^{^{\prime} }$ and where

3 $${T}_{i}^{^{\prime} }={T}_{i}^{^{\prime} }({\theta }_{i},{a}_{i},{d}_{i},-\,(\pi -{\alpha }_{i}))$$

4 $${T}_{i+1}^{^{\prime} }={T}_{i+1}^{^{\prime} }(\,-\,{\theta }_{i+1},{a}_{i+1},-\,{d}_{i+1},-\,(\pi -{\alpha }_{i+1}))$$

*then* $${T}_{i}{T}_{i+1}={T}_{i}^{^{\prime} }{T}_{i+1}^{^{\prime} }$$.

#### **Corollary 1.1**

*Let B be a hooplinkage as described in Definition 1*. *If a hinge is transposed as defined in Theorem 1*, *the new mechanism B*′ *will also be a hoop linkage*. *Also*, *it will continue to be capable of motion*.

Theorem 1 and Corollary 1.1 have important consequences. Together, they describe a simple process for modifying the geometry of an origami-inspired linkage without affecting its motion. One application of this modification would be to reduce self-intersection.

### Thickness Shift

**Figure 3:**
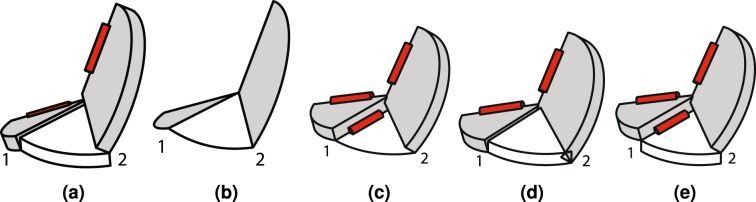
Thickness shift. Three links where (**a**) thicknesses are non-zero, (**b**) *a*_1_ = *a*_2_ = *a*_3_ = 0 (spherical mechanism), (**c**) *a*_2_ = 0, (**d**) *a*_2_ = 0 and the panel thickness is above and (**e**) *a*_2_ = 0 and the panel thickness is below.

Here, we introduce another modification method called thickness shift which requires that *a*_*i*_ = 0 for a certain subset of the transforms *T*_1_*T*_2_ … *T*_*n*_. This operation is illustrated in Fig. 3. If the thickness shift is performed on all the links, then we obtain the spherical mechanism shown in Fig. 3b. However, if it is only applied to the middle link, then Fig. 3c shows the resulting mechanism. While the middle link may be drawn with no thickness, it may also include the material thickness above or below the panel as illustrated in Fig. 3d,e. Unlike the hinge transpose, this type of transformation does not necessarily result in an equivalent linkage. For a hoop linkage, changing the thickness parameter *a* to zero must respect any additional constraints enforced by the loop. For the Bennett linkage, these requirements are shown in SI Equations 2, 3 and 7. For this particular linkage, these equations can only be satisfied if all thicknesses are zero. Consequently, a thickness shift transformation can only be applied to either all or none of the links. Similar rules will apply to other linkage types.

### Split Vertex

**Figure 4:**
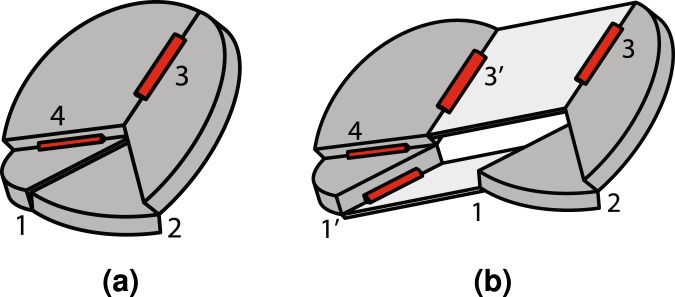
Split vertex. (**a**) An initial vertex which is (**b**) split with two offset panels.

A final modification that we consider is the split vertex technique^14,37^. In this technique, two links representing a linear offset are included at two arbitrary, non-consecutive points in a hoop linkage consisting of *n* links. This transformation is shown in Fig. 4. This modification is possible as long as a vector from 3 to 3′ and 1 to 1′ are identical and hinge 3 is parallel to 3′ and hinge 1 is parallel to 1′. The fact that this modification also preserves the ability of the hoop linkage to move can also be proven (see the corresponding proof in the SI).

#### **Theorem 2**

*Let B be a hoop linkage as described in Definition 1*, ***d***^*i*^ *represent a translation with respect to frame i and T*_*i*_ *and T*_*j*_ *be homogeneous transformations where*

5 $${T}_{i}=(\begin{array}{cc}{\boldsymbol{I}} & {{\boldsymbol{d}}}^{i}\\ {\boldsymbol{0}} & 1\end{array})$$

6 $${T}_{j}=(\begin{array}{cc}{\boldsymbol{I}} & -{{\boldsymbol{d}}}^{j}\\ {\boldsymbol{0}} & 1\end{array})$$

*For the homogeneous transformations describing B*, *T*_1_*T*_2_ … *T*_*n*_ = *T*_1_*T*_2_ … *T*_*i*−1_*T*_*i*_*T*_*i*+1_ … *T*_*n*_*T*_*j*_ = *I and the new set of links is also a hoop linkage as described by Definition 1*.

This result is significant because it shows that a split vertex transformation can be applied to any non-consecutive hinges in a hoop linkage provided the offset links *T*_*i*_ and *T*_*j*_ have certain properties: they must both be linear translations described by vector **d** and the hinges that they connect to must be parallel because *T*_*i*_ and *T*_*j*_ include the identity rotation **I**.

### Linkage Permutations

**Figure 5:**
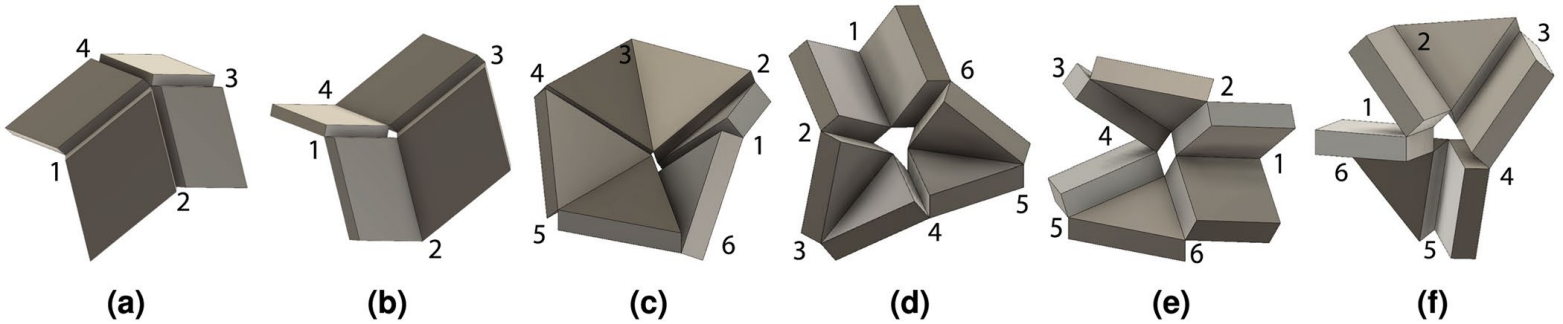
Linkage variations. Linkages (**a**) II and (**b**) III are variations of the Bennett linkage and linkages (**d**) IV, (**e**) V and (**f**) VI are variations of the Bricard linkage.

The hinge transpose, thickness shift and split-vertex methods can be used to generate variations of a particular hoop linkage. If we designate a hinge transpose by *T*[*u*_*t*_], a thickness shift by *Z*[*u*_*z*_] and a vertex split by *V*[*u*_1_, *u*_2_], then recording alterations can be simplified. In this notation, *u*_*t*_ is the hinge that is transposed, *u*_*z*_ is the smallest hinge number adjacent to the thickness shift and *u*_1_ and *u*_2_ are the lowest-numbered hinges adjacent to the offset links in the split mechanism. If we list the left-most operation occurring first, a table can be created that connects every variation to a fundamental set of hoop linkages. This information is listed in Table 1. For *n* = 4, the Bennett linkage described in Fig. 2a is used as a starting point. For *n* = 6, a vertex in Fig. 5c that was described by Chen *et al*.^18^ is the starting point.

**Table 1:** A list of the number of links, *n*, in each linkage, the figure showing the final linkage, the transformations required to create each derivative linkage from its starting form and whether the final linkage is developable (D), over- (O) or under-developed (U).

Name	*n*	Figure	Transformations	Σ*α*
Linkage I	4	2c	*T*[4]	D
Linkage II	4	5a	*T*[4], *T*[1], *Z*[*all*]	U
Linkage III	4	5b	*T*[4], *T*[1]	O
Linkage IV	6	5d	*T*[1]	O
Linkage V	6	5e	*T*[1], *T*[4], *Z*[2], *Z*[5]	O
Linkage VI	6	5f	*T*[1], *T*[4]	O
Linkage VII	6	4b	T[4]V[1,3]	D

The 4-link and 6-link models are derived from the root models in Figs 2a and 5c, respectively.

Different linkage variations created by the hinge transpose and thickness shift methods can also be combined to form more complex mechanisms. An example of a model using Linkages I and III is shown in Fig. S1 and Video S2. The hinges for this model are shown in Fig. S2. In this combined form, the new mechanism acts like an extendable tube.

### Tessellation of Linkage-Based Origami

**Figure 6:**
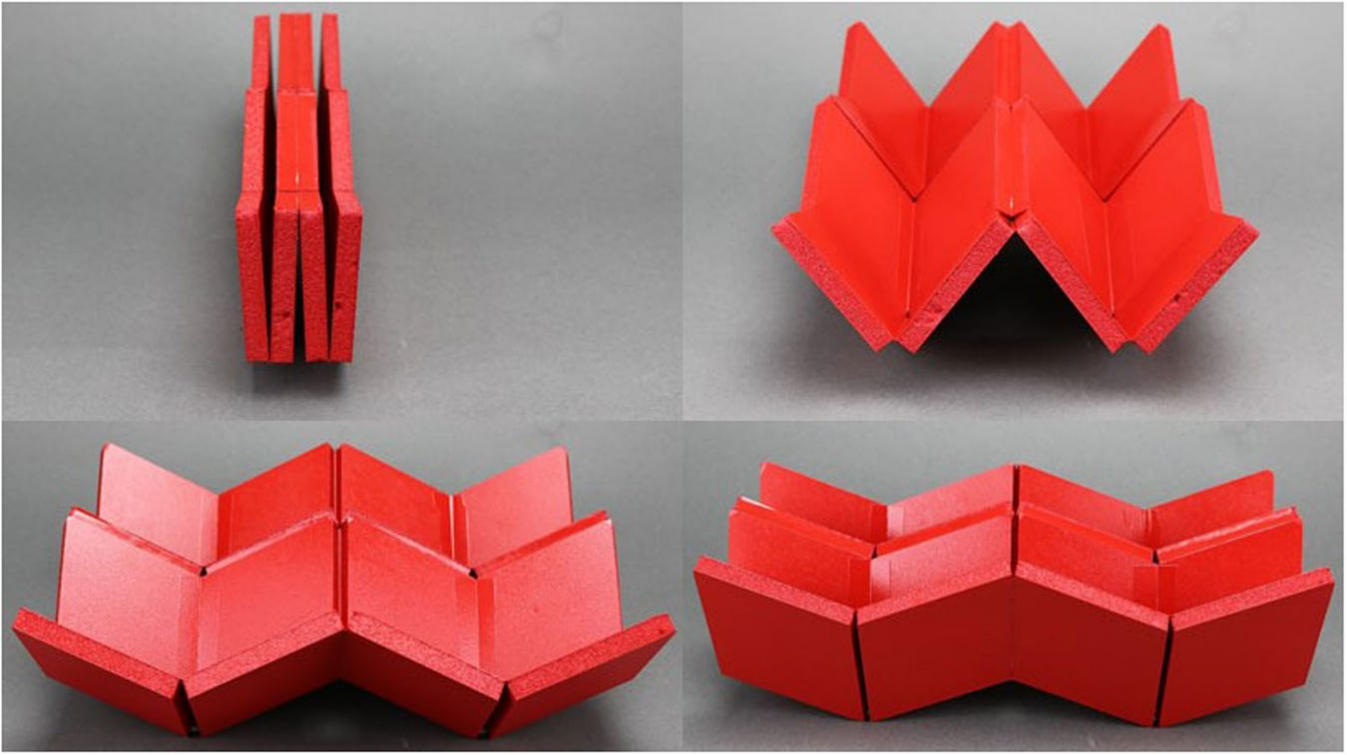
2D tessellation. A basic Eggbox tessellation composed of Linkage II and III vertices.

The ability of linkage-based origami to tessellate in two dimensions is an important characteristic that will influence its suitability for engineering applications such as large deployables. Such a tessellation is possible using developable vertices^27,38,39^, but complicated geometry is required to prevent self-intersection in the folded state for thick models. Tessellation is also possible using the thick, Eggbox tessellation shown in Fig. 6. The combination of Linkage II and III allows the entire pattern to fold without self-intersection. While this pattern is useful in its current state, understanding how the geometric constraints in the mechanism influence its tailorability enable its deployment to be tailored to specific applications.

Connecting hoop linkages in a network and ensuring that the network is rigid-foldable is similar to ensuring rigid-foldability in zero-thickness origami networks because a similar set of constraints apply. One of these is that fold angles must be consistent when traced in loops containing multiple vertices and creases^40^. Another constraint results from the need for panel thicknesses to be compatible throughout the pattern. While it is possible to allow discontinuities in the panel thickness, this will not be considered here. With this information, a set of equations describing this specific tessellation can be found.

While criteria for designing networks of rigid-foldable linkages have not been mathematically proved, it will be assumed that satisfying three different constraint types throughout the pattern will be sufficient. These constraints are: Thickness Compatibility, Fold-Angle Compatibility and Sector-Angle Compatibility.

#### Thickness Compatibility

**Figure 7:**
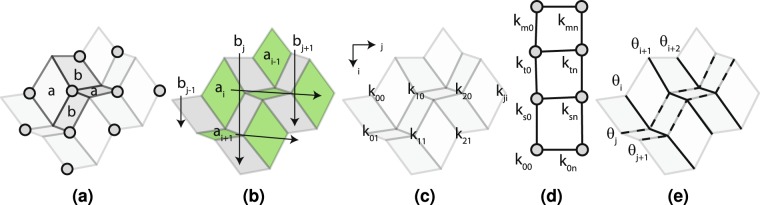
Tessellation geometry. The constraints introduced in a linkage tessellation when thickness must be compatible. (**a**) Opposite panels around a vertex with equal thickness. (**b**) Thickness constraints in a linkage tessellation. (**c**) Vertex *k*-constants. (**d**) Multiple constraint loops. (**e**) Creases that must have equal fold angles.

One constraint affecting Linkage III is that opposite panels must have the same thickness, as shown in Fig. 7a. In this figure, opposite panels around the vertices marked with dots have the same thickness *a* or *b*. Extending this constraint across the pattern requires that panels along diagonals be of the same thickness *a*_*i*_ or *b*_*j*_ as shown in Fig. 7b.

A relationship based on specific thickness ratios determined by the twist angles provides an additional loop thickness constraint. Specifically, this relation requires that

7 $$\frac{a}{b}=\frac{\sin \,{\alpha }_{1}}{\sin \,{\alpha }_{2}}=\frac{1}{k}$$

where *k* is the thickness ratio *b*/*a*. The loop constraints created by Equation 7 become clear when we consider that a line of constant thickness *a*_*i*_ is related to perpendicular lines of constant thickness *b*_*j*_ by the relation *b*_*j*_ = *k*_*ji*_*a*_*i*_ where *k*_*ji*_ is a thickness ratio calculated for a particular vertex at coordinates *j* and *i* as shown in Fig. 7c. If we relate a given thickness line *a*_0_ to its perpendicular at coordinates (*j*, *i*) = (0, 0), then we have *b*_0_ = *k*_00_*a*_0_. If this thickness is related to a thickness line parallel to *a*_0_ at some coordinate (0, *n*), then we have

8 $${a}_{n}=\frac{{b}_{0}}{{k}_{0n}}=\frac{{k}_{00}}{{k}_{0n}}{a}_{0}$$

Similarly, this thickness line can be related to its perpendicular line at coordinates (*m*, *n*). This gives

9 $${b}_{m}={k}_{mn}{a}_{n}={k}_{mn}(\frac{{k}_{00}}{{k}_{0n}}{a}_{0})=\frac{{k}_{mn}{k}_{00}}{{k}_{0n}}{a}_{0}$$

Finally, this thickness can be related to the original thickness line, *a*_0_. This gives

10 $${a}_{0}=\frac{{b}_{m}}{{k}_{m0}}=\frac{{k}_{mn}{k}_{00}}{{k}_{m0}{k}_{0n}}{a}_{0}$$

Assuming that *a*_0_ ≠ 0 allows it to be eliminated from both sides of Equation 10, resulting in

11 $${k}_{mn}=\frac{{k}_{m0}{k}_{0n}}{{k}_{00}}$$

This equation must hold for any loop of Linkage III vertices. However, listing all possible loops may be difficult if the pattern is large. The number of equations can be reduced if we recognize that knowing that loop (*k*_00_, *k*_0*n*_, *k*_*sn*_, *k*_*s*0_) and (*k*_00_, *k*_0*n*_, *k*_*tn*_, *k*_*t*0_) are satisfied guarantees that loop (*k*_*s*0_, *k*_*sn*_, *k*_*tn*_, *k*_*t*0_) is also satisfied. This situation is depicted in Fig. 7d. Here, we assume that 0 < *i* < *m*. This can be shown by expressing thickness consistency using Equation 11 for both loops. This yields

12 $${k}_{00}=\frac{{k}_{s0}{k}_{0n}}{{k}_{in}}$$

13 $${k}_{tn}{k}_{00}={k}_{t0}{k}_{0n}$$

Substituting Equations 12 into 13 and eliminating *k*_00_ gives *k*_*tn*_*k*_*s*0_ = *k*_*t*0_*k*_*sn*_ which matches the condition for the third loop (*k*_*s*0_, *k*_*sn*_, *k*_*tn*_, *k*_*t*0_). Generally, consistency of all interior loops can be shown by guaranteeing that all loops (*k*_00_, *k*_0*i*_, *k*_*ji*_, *k*_*j*0_) are compatible for all *j* ∈ {1, 2, 3 … *m*} and *i* ∈ {1, 2, 3 … *n*}.

Equation 13 is significant because it suggests that specifying *k*_00_, *k*_*m*0_ and *k*_0*n*_ will determine all *k*_*mn*_ in the interior of the pattern. In the context of pattern design, this indicates that setting the vertex panel angles *α*_1_ and *α*_2_ for all the Bennett linkages along two edges of a rectangular pattern should specify all *k*_*i*_ elsewhere in the pattern. While the panel angles for these interior vertices will not be completely determined, thickness consistency loops will provide additional constraints on the pattern geometry.

#### Fold-Angle Compatibility

A second set of constraints derives from maintaining compatible fold angles between all vertices in the tessellation because they share hinges. Making the substitutions required by Table 1 in SI Equations 8 through 10 shows that 2*π* = −*θ*_1_ + *θ*_3_, 2*π* = *θ*_2_ − *θ*_4_. These relations are equivalent to *θ*_1_ = *θ*_3_ and *θ*_2_ = *θ*_4_ if we assume that *θ*_*i*_ = *θ*_*i*_ + 2*π*. If opposite fold angles in a vertex are equal, then the opposite-angle chains shown in Fig. 7e should all be of equal angles. These chains are indicated by uniform line types and angles *θ*_*i*_, *θ*_*i*+1_ and *θ*_*i*+2_. The relationship between the angles in Linkages II and III can be found by making the appropriate substitutions in SI Equation 10, giving

14 $$\tan \,\frac{{\theta }_{i}}{2}\,\tan \,\frac{{\theta }_{j}}{2}=\frac{\cos \,\frac{1}{2}({\alpha }_{1}-{\alpha }_{2})}{\cos \,\frac{1}{2}({\alpha }_{1}+{\alpha }_{2})}={\mu }_{ji}$$

Like Evans, *et al*.^40^ we define the constant *μ* to simplify calculations. Here, *μ*_*ji*_ is calculated for a vertex with coordinates *i* and *j* similar to those shown in Fig. 7c. Like constraints on thickness, perpendicular lines of constant fold angle can be related in loops between four vertices (0, 0),(0, *n*),(*m*, *n*) and (*m*, 0). This relationship can be expressed as

15 $${\mu }_{mn}=\frac{{\mu }_{m0}{\mu }_{0n}}{{\mu }_{00}}$$

Because this equation has the same form as Equation 11, it can be generalized in the same way. When combined, these two equations fill a similar roll as those in^27^ and manage constraint in this linkage tessellation.

#### Sector-Angle Compatibility

The final set of constraints guarantee that the panels of the linkage tessellation are quadrilaterals and is written as

16 $$2\pi ={\alpha }_{ji}+{\alpha }_{j+1,i}+{\alpha }_{j+1,i+1}+{\alpha }_{j,i+1}$$

However, in its current form this equation cannot be easily integrated with the other equations. An assumption that will facilitate integration is that panel thickness is uniform throughout the pattern. This assumption will be desirable for manufacturing considerations but will also simplify the resulting equations. If *a*/*b* = 1, then sin *α*_1_ = sin *α*_2_. Satisfying this equality requires that either *α*_1_ = *α*_2_ or *α*_2_ = *π* − *α*_1_. The latter is impossible for non-developable vertices since for the angle sum Σ*α* = 2*α*_1_ + 2(*π* − *α*_2_) ≠ 2*π*. If *α*_1_ = *α*_2_, then Equation 15 evaluated for a single panel reduces to sec *α*_*j*+1,*i*+1_ sec *α*_*ji*_ = sec *α*_*j*+1,*i*_ sec *α*_*j*,*i*+1_. Equation 16 can be used to eliminate *α*_*j*+1,*i*+1_ and show that

17 $$\begin{array}{rcl}\cos \,({\alpha }_{ji}+{\alpha }_{j+1,i}+{\alpha }_{j,i+1})\,\cos \,{\alpha }_{ji} & = & \cos \,{\alpha }_{j+1,i}\,\cos \,{\alpha }_{j,i+1}\\ \sin \,({\alpha }_{ji}+{\alpha }_{j+1,i})\,\sin \,({\alpha }_{ji}+{\alpha }_{j,i+1}) & = & 0\end{array}$$

Equation 17 can only be satisfied if either *α*_*ji*_ + *α*_*j*,*i*+1_ = 0, *π* or *α*_*ji*_ + *α*_*j*+1,*i*_ = 0, *π*. Because *α*_*ji*_ > 0, the angle sums must equal *π*. These relations effectively eliminate control over either all the vertices (0, *n*) or (*m*, 0). Once the geometry of either the horizontal or vertical row of vertices are set, the perpendicular direction is also defined.

## Discussion

Linkage-based origami-inspired mechanisms have the potential to solve difficult engineering problems because of their unique ability to realize rigid-foldable mechanisms as well as incorporate materials with finite thickness. These two traits can be valuable in applications such as deployable equipment for spacecraft. However, important challenges have complicated the design of these mechanisms.

This work demonstrates a method for generating mechanism variations that can result in new thick-origami designs. A mathematical basis for generating different variations of an overconstrained, hoop linkage was proved and shown to produce legitimate variations of a hoop linkage. This was demonstrated by creating six variations of linkage-based origami vertices based on the Bennett and Bricard linkages. Equations describing the kinematics of these mechanisms were also derived to characterize the deployment of the linkage tessellation.

**Figure 8:**
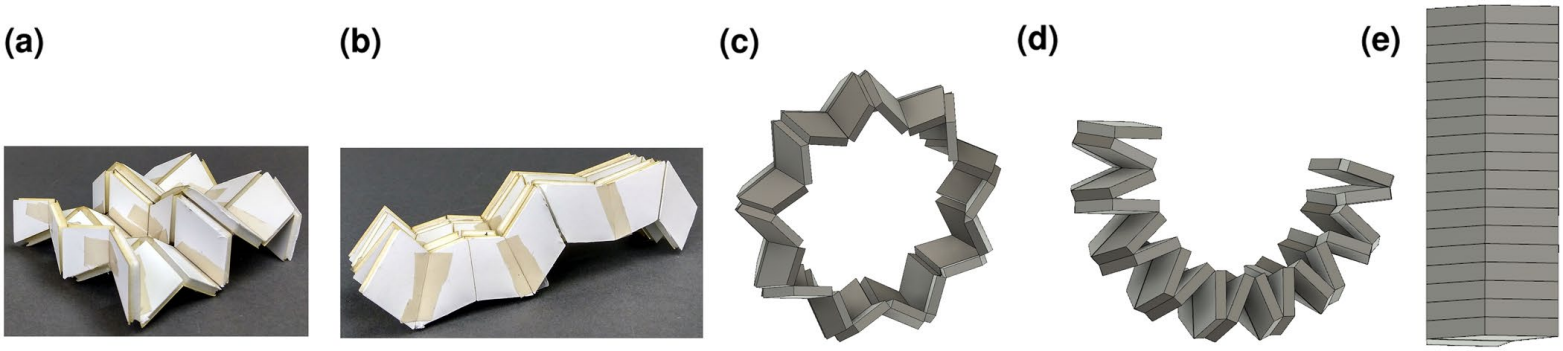
Curved tessellation. A model of a linkage tessellation (**a**) with a sinusoidal profile, one of its stowed states (**b**) similar to the original Eggbox and a wheel (**c**) which can be folded through state (**d**) to state (**e**) using the curved Linkage III mechanism.

Another challenge facing linkage-based origami design is ensuring that they are rigid-foldable. While relations for designing thin, quadrilateral-mesh origami have been developed^27^, designing thick versions has proven challenging. This paper demonstrated that in some cases it is acceptable to require only these three classes of constraints to design rigid-foldable networks of linkage-based vertices. For patterns of quadrilateral panels and fourth-order vertices like the linkage tessellation, a method for ensuring that these constraints were met throughout the pattern was derived and demonstrated to allow design of the deployed mechanism shape. This method was used to design the mechanisms shown in Fig. 8a. Details on its construction and a video of its motion (Video S1) are provided as supporting information.

A potential application for this method is the design of deployable, curved structures such as rover wheels^41^. Deployable wheels for rovers are desirable because space is limited in a lander and reducing wheel size can save significant amounts of volume^42^. Another advantage of wheel deployment is the increased cross-country ability that larger wheels can provide^43^. A wheel approximated by a linkage tessellation of constant curvature is shown in Figs 8c through 8e. Details on its construction are provided as supporting information. This particular model is capable of folding a 15 centimeter diameter wheel into approximately one sixth the original volume. Because of the method used, the design also has significant design flexibility. If wider wheels are desired, additional rows can be added. If the wheel profile needs to be more or less jagged, the vertex geometry can be adjusted and vertices can be added or removed.

Addressing the challenges of accommodating thickness and ensuring rigid-foldability will enable novel deployable mechanisms because of the solution simplicity. It is hoped that the increased accessibility of methods for developing variations of overconstrained linkages will enable knowledge in origami to be exploited to create systems not previously possible.

## Methods

### Model Construction

The model in Fig. 8a was constructed using vertices with angles shown in the upper rows of Table 2. The model in Fig. 8c was fabricated using vertices with the vertex angles shown in the lower rows of Table 2. No datasets were generated or analyzed during the current study.

**Table 2:** The panel sector angles in degrees for each vertex in the prototypes in Fig. 8a,c are listed for each row *i* and column *j* location of the vertex.

	*j*=	1	2	3	4	5	6	…	17	18
Fig. 8a	*i* = 1	120°	60°	120°	60°	110°	60°	—	—	—
	*i* = 2	60°	120°	60°	120°	80°	120°	—	—	—
	*i* = 3	120°	60°	120°	60°	110°	60°	—	—	—
Fig. 8c	*i* = 1	100°	70°	100°	70°	100°	70°	…	100°	70°

## Electronic supplementary material

Video S1

Video S2

Supplementary information
