# Supplementary material for: Creating Linkage Permutations to Prevent Self-Intersection and Enable Deployable Networks of Thick-Origami
Source: Sci Rep. 2018 Aug 28;8:12936. doi: 10.1038/s41598-018-31180-4 (PMC6113247; doi:10.1038/s41598-018-31180-4)
Supplement: Supplementary file 3 — Supplementary information [file 41598_2018_31180_MOESM3_ESM.pdf]

Supplementary Information: Creating Linkage Permutations to Prevent Self-Intersection and Enable Deployable Networks of Thick-Origami

Alden Yellowhorse[1], Robert J. Lang[2], Kyler Tolman[3], and Larry L. Howell[1,*]

[1]Dept. Mechanical Engineering, Brigham Young University, Provo, UT 84602
[2]Lang Origami, Alamo, CA 94507
[3]Toyota Motor North America, Saline, MI 48176
[*]To whom correspondence should be addressed. E-mail: lhowell@byu.edu

Homogeneous Transformation

The homogeneous transformation T_i is given by

$$T_i = \begin{pmatrix} \mathbf{R} & \mathbf{d} \\ \mathbf{0} & 1 \end{pmatrix} = \begin{pmatrix} \mathrm{C}(\theta_i) & -\mathrm{C}(\alpha_i)\,\mathrm{S}(\theta_i) & \mathrm{S}(\alpha_i)\,\mathrm{S}(\theta_i) & \mathrm{C}(\theta_i)\,a_i \\ \mathrm{S}(\theta_i) & \mathrm{C}(\alpha_i)\,\mathrm{C}(\theta_i) & -\mathrm{C}(\theta_i)\,\mathrm{S}(\alpha_i) & \mathrm{S}(\theta_i)\,a_i \\ 0 & \mathrm{S}(\alpha_i) & \mathrm{C}(\alpha_i) & d_i \\ 0 & 0 & 0 & 1 \end{pmatrix} \tag{1}$$

where $\mathbf{R}$ is a 3×3 rotation matrix, $\mathbf{d}$ is a 3×1 translation vector, $C(...) = \cos(...)$ and $S(...) = \sin(...)$.

Bennett Linkage Relationships

It has been shown[1] that Definition 1 results in the following equations for a mechanism with four links:

$$a_1 = a_3 \tag{2}$$
$$a_2 = a_4 \tag{3}$$
$$\alpha_1 = \alpha_3 \tag{4}$$
$$\alpha_2 = \alpha_4 \tag{5}$$
$$d_1 = d_2 = d_3 = d_4 = 0 \tag{6}$$
$$\frac{a_1}{a_2} = \frac{\sin\alpha_1}{\sin\alpha_2} \tag{7}$$
$$2\pi = \theta_1 + \theta_3 \tag{8}$$
$$2\pi = \theta_2 + \theta_4 \tag{9}$$
$$\tan\frac{\theta_1}{2}\tan\frac{\theta_2}{2} = \frac{\sin(\frac{1}{2}(\alpha_2+\alpha_1))}{\sin(\frac{1}{2}(\alpha_2-\alpha_1))} \tag{10}$$

where θ_i, a_i, d_i, and α_i correspond to the DH parameters defined previously. An interesting observation that can be made about Equation 10 is its similarity to the fold angle multiplier μ defined for rigid-foldable origami[2].

Proofs

Proof of Theorem 1

Proof. Showing equivalence is possible by making the substitutions below:

$$\alpha_j = -(\pi - \alpha'_j) \tag{11}$$
$$\alpha_k = -(\pi - \alpha'_k) \tag{12}$$
$$\theta_k = -\theta'_k \tag{13}$$
$$d_k = -d'_k \tag{14}$$

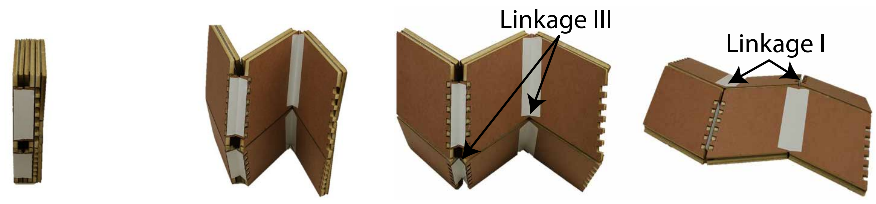

(a) **(b)**

Figure S1. Example thick-origami tube. Foam-board prototype of a non-developable-thick tube. Its motion is further illustrated in Video S2.

and the transformations become

$$T_1' = \begin{pmatrix} C(\theta_j) & C(\alpha_j)S(\theta_j) & -S(\alpha_j)S(\theta_j) & C(\theta_j)a_j \\ S(\theta_j) & -C(\alpha_j)C(\theta_j) & C(\theta_j)S(\alpha_j) & S(\theta_j)a_j \\ 0 & -S(\alpha_j) & -C(\alpha_j) & d_j \\ 0 & 0 & 0 & 1 \end{pmatrix} \tag{15}$$

$$T_2' = \begin{pmatrix} C(\theta_k) & -C(\alpha_k)S(\theta_k) & S(\alpha_k)S(\theta_k) & C(\theta_k)a_k \\ -S(\theta_k) & -C(\alpha_k)C(\theta_k) & C(\theta_k)S(\alpha_k) & -S(\theta_k)a_k \\ 0 & -S(\alpha_k) & -C(\alpha_k) & -d_k \\ 0 & 0 & 0 & 1 \end{pmatrix} \tag{16}$$

Taking the difference between the two pairs of transformations and simplifying yields

$$T_1'T_2' - T_1T_2 = 0 \tag{17}$$

Therefore, $T_1'T_2' = T_1T_2$, □

Proof of Corollary 1.1

Proof. By Definition 1, a closed linkage is a linkage if $T_1T_2...T_n = I$. Also, Theorem 1 guarantees that $T_iT_{i+1} = T_i'T_{i+1}'$. Without loss of generality, making this substitution in the linkage relation yields $T_1'T_2'T_3...T_n = I$. Because T_1' and T_2' are also homogeneous transformations, the modified linkage is still a linkage. □

Proof of Theorem 2

Proof. Because the product of any number of homogeneous transformations is also a homogeneous transformation, we can write $T_1T_2...T_{i-1}T_iT_{i+1}...T_nT_j = T_aT_iT_bT_j$. Evaluating the product $T_iT_bT_j$ gives

$$\begin{pmatrix} \mathbf{I} & \mathbf{d}^i \\ \mathbf{0} & 1 \end{pmatrix} \begin{pmatrix} \mathbf{R}_b & \mathbf{d}_b^{i+1} \\ \mathbf{0} & 1 \end{pmatrix} \begin{pmatrix} \mathbf{I} & -\mathbf{d}^j \\ \mathbf{0} & 1 \end{pmatrix} = \begin{pmatrix} \mathbf{R}_b & -\mathbf{R}_b\mathbf{d}^j + \mathbf{d}_b + \mathbf{d}^i \\ \mathbf{0} & 1 \end{pmatrix} \tag{18}$$

Because $\mathbf{R}_b$ relates the frame at j to the frame at i, we know that $\mathbf{d}^i = \mathbf{R}_b\mathbf{d}^j$. Substituting this relation into Equation 18 shows that $T_iT_bT_j = T_b$. Therefore, $T_1T_2...T_n = T_1T_2...T_{i-1}T_iT_{i+1}...T_nT_j = I$. Also, because the new product of transformations is equal to the identity transformation, it is also a linkage. □

Other Multi-Vertex Mechanisms

Models constructed with Linkage I and II vertices are shown in Figures S1 and S2. Figure S3 shows the linkages used to construct the model in Figure S1.

Prototype Geometry

The prototypes in Fig. 8 in the main text were designed using several specific formulas derived using the methods presented in the paper. Equations 11, 15 and 17 can be used to design linkage tessellations with controllable shape by specifying the number of available degrees of freedom in a given model. One procedure for setting these degrees of freedom is by specifying a zig-zag pattern as shown in Fig. S4a that sufficiently approximates a desired curve. Because this curve approximation sets

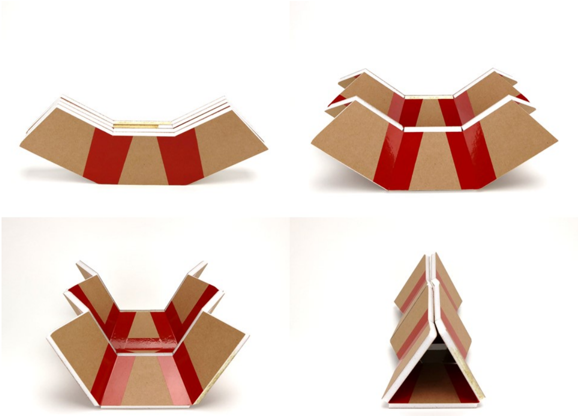

Figure S2. Alternate tube design. Eggbox inspired non-developable tube comprised of excess-vertex Bennett linkages and deficient-vertex spherical linkages.

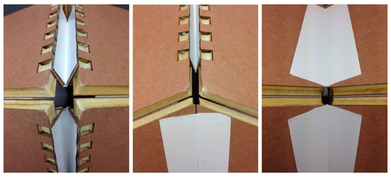

Figure S3. Linkages used in tube. The two different linkages used in the symmetric-thick-origami tube. Left: Linkage III. Center: Linkage I. Right: Linkage III

vertex geometry along a row of vertices with coordinates $(0,n)$, the relationships defined above specify the geometry of the remaining pattern. If we specify the individual segment lengths $L_1, L_2 ... L_n$, the internal angles $\phi_1, \phi_2 ... \phi_n$, the starting fold angle θ_i and assume that $\alpha_1 = \alpha_2$, the remaining parameters can be found. Given θ_i and all L's and ϕ's, we have

$$\tan\frac{\theta_i}{2}\tan\frac{\theta_j}{2} = \mu_{ji} = \sec\alpha_{ji} \tag{19}$$

The spherical law of cosines can be used to relate α_{ji}, θ_j and ϕ_i in Fig. S4, giving

$$\cos\phi_i = \cos^2\alpha_{ji} + \sin^2\alpha_{ji}\cos\theta_j = \cos^2\alpha_{ji} + (1-\cos^2\alpha_{ji})\cos\theta_j = \frac{1-\cos\theta_j}{\sec^2\alpha_{ji}} + \cos\theta_j = \frac{1-\cos\theta_j}{\tan^2\frac{\theta_i}{2}\tan^2\frac{\theta_j}{2}} + \cos\theta_j$$

$$\cos\theta_j = -\frac{1}{2}(1+\cos\theta_i) + \cos\phi_i\sin^2\frac{\theta_i}{2} \tag{20}$$

If θ_i and ϕ_i are known, then θ_j can be determined from Equation 20. This can in turn be used to calculate α_{ji} from Equation 19. The exact values of L_i can be set freely. Because the geometry expressed in Fig. S4 applies to both over- and under-developed vertices, this procedure can be used to design both types of vertices.

Vertex Developability

Here, we define developability and related terms. These new mechanism configurations can be categorized based on the sum of all the panel angles, D, where

$$D = \sum_i^n |\alpha_i| \tag{21}$$

and α_i are the panel angles for a given mechanism. If $D < 2\pi$, then the vertex is defined as under-developed. If $D = 2\pi$, then the vertex is developable. If $D > 2\pi$ then the vertex is defined as over-developed.

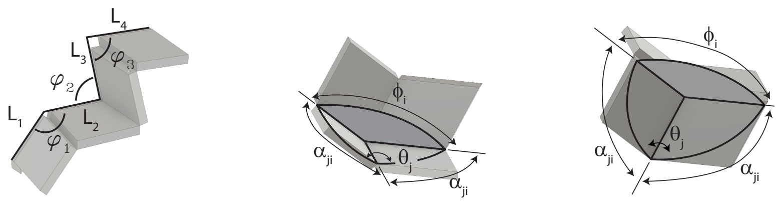

(a) Both Linkages **(b)** Linkage II **(c)** Linkage III

Figure S4. Variables defining tessellation geometry. (a) A zig-zag profile formed by the ridge of the pattern described by angles ϕ_i and lengths L_i and (b) (c) dimensions used in the analysis of both Linkage II an III vertices

Author contributions statement

A.Y. and K.T. developed initial content. A.Y., R.L. and L.H. revised content and refined final draft.

References

1. Chen, Y. & You, Z. Mobile assemblies based on the bennett linkage. *Proc. Royal Soc. Lond. A: Math. Phys. Eng. Sci.* **461**, 1229–1245 (2005). DOI 10.1098/rspa.2004.1383.

2. Evans, T. A., Lang, R. J., Magleby, S. P. & Howell, L. L. Rigidly foldable origami gadgets and tessellations. *Royal Soc. Open Sci.* **2** (2015). DOI 10.1098/rsos.150067.

Figure S1. Example thick-origami tube. Foam-board prototype of a non-developable-thick tube. Its motion is further illustrated in Video S2.

Figure S2. Alternate tube design. Eggbox inspired non-developable tube comprised of excess-vertex Bennett linkages and deficient-vertex spherical linkages.

Figure S3. Linkages used in tube. The two different linkages used in the symmetric-thick-origami tube. Left: Linkage III. Center: Linkage I. Right: Linkage III

Figure S4. Variables defining tessellation geometry. (a) A zig-zag profile formed by the ridge of the pattern described by angles ϕ_i and lengths L_i and (b) (c) dimensions used in the analysis of both Linkage II an III vertices
